# Carcinoid Heart Disease: A Rare Complication of Metastatic Neuroendocrine Tumor

**DOI:** 10.7759/cureus.78148

**Published:** 2025-01-28

**Authors:** John P Martinez Ponce, Oskar Ubysz, Thomas Vanhecke

**Affiliations:** 1 Internal Medicine, Henry Ford Health, Grand Blanc, USA; 2 Cardiology, Henry Ford Health, Grand Blanc, USA

**Keywords:** carcinoid heart disease, heart failure, liver metastasis, neuroendocrine cell tumor, serotonin receptor

## Abstract

Carcinoid heart disease (CHD), also known as Hedinger syndrome, is a rare but significant cardiac complication associated with metastatic neuroendocrine tumors (NETs). These tumors secret bioactive substances such as serotonin, leading to fibrotic changes primarily affecting the right-sided heart valves. A case study involving a 69-year-old male presented with a four-month history of diarrhea and a new systolic heart murmur. Transthoracic echocardiogram (TTE) results indicated a left ventricular ejection fraction (LVEF) of 60% to 65%, with severe tricuspid regurgitation and pulmonary valve stenosis. Elevated levels of 5-hydroxyindoleacetic acid (5-HIAA) were detected in a 24-hour urine test, and imaging revealed multiple hypoechoic masses in the liver and mesenteric masses adherent to the small intestine. Furthermore, a biopsy confirmed the diagnosis of a NET. Medical therapy like long-acting somatostatin injection and a peptide receptor radionuclide therapy was ineffective in reversing established valvular pathology, and the patient continued to experience clinical decline, suffering from right-sided heart failure. The patient was able to undergo combined tricuspid and pulmonary valve replacement, which resolved his symptoms. This case exemplifies the successful treatment of a rare syndrome leading to right heart failure.

## Introduction

Carcinoid heart disease (CHD) is a cardiac complication of metastatic neuroendocrine tumors (NETs), such as carcinoid tumors, and can result in severe heart failure symptoms. NETs secrete up to 40 different chemical substances that contribute to various clinical symptoms, including chronic diarrhea, dyspnea, shortness of breath, cutaneous flushing, fibrosis (pulmonary, mesenteric, or retroperitoneal), bronchospasm, gastrointestinal bleeding, and bowel obstruction-induced constipation [1]. Carcinoid tumors are rare, with an incidence of 5.25 per 100,000, arising primarily in the gastrointestinal tract (67.5%) and bronchopulmonary system (25.3%) but also in tissues such as the pancreas and ovaries [2]. Despite their rarity, 56% of patients with these tumors develop cardiac dysfunction, particularly in the presence of hepatic metastases, which allow serotonin and tachykinins to bypass hepatic metabolism and affect cardiac tissues [3]. These bioactive substances exert fibrotic effects on the heart valve endocardium, leading to CHD, predominantly characterized by right-sided valvular dysfunction involving the tricuspid and pulmonary valves [3]. Pulmonary filtration of these substances protects left-sided heart valves, making right-sided valve damage more common.

For instance, Mohd Nasri et al. emphasized the progressive nature of CHD, which underscores the necessity for early and regular echocardiographic screening in patients with known carcinoid syndrome [4]. Similarly, Duijnhouwer et al. demonstrated that isolated tricuspid valve involvement could be the initial indication of CHD in some cases, highlighting the disease's heterogeneity [5]. A multidisciplinary approach has been recommended for managing these complex cases, as evidenced by recent studies exploring surgical and emerging pharmacological interventions [6,7]. Moreover, advanced imaging modalities like cardiac MRI, Echocardiography, cardiovascular CT, and positron emission tomography (PET) have shown significant promise in assessing myocardial function and viability, further enhancing the ability to tailor treatments to the individual needs of patients with CHD [8].

The following case explores a patient whose progression of CHD aligns with these findings, advancing from initial asymptomatic murmurs to severe valvular dysfunction. Through this case, we aim to underscore the clinical importance of timely diagnosis and highlight the therapeutic challenges associated with CHD.

## Case presentation

A 69-year-old male presented with a four-month history of chronic diarrhea and was referred by his primary care provider for evaluation of a newly detected murmur, as well as findings from a recent transthoracic echocardiogram (TTE). The patient had no prior history of cardiac disease and denied symptoms such as shortness of breath, chest pain, palpitations, or lower extremity edema at the time of presentation. On physical examination, prominent jugular pulsation and a loud pulmonic systolic ejection murmur were noted.

The initial TTE revealed a left ventricular ejection fraction (LVEF) of 60-65% with normal global systolic function and no diastolic dysfunction. However, septal flattening was observed during diastole, suggesting right ventricular pressure overload. While the left atrium appeared normal, the right ventricle was significantly dilated with moderately reduced functionality. The tricuspid valve annulus was severely dilated, and the tricuspid valve leaflets were fixed in an open position (Video 1 and Video 2). Severe tricuspid regurgitation was noted.

**Video 1:** Apical four-chamber view of fibrosed and immobile tricuspid leaflets.

**Video 2:** Parasternal short axis view, showing severe tricuspid regurgitation.

**Figure 1:**
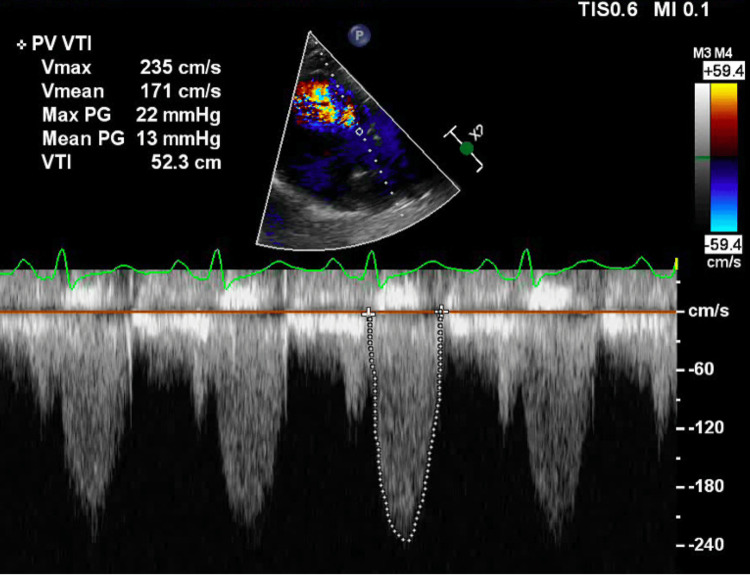
Parasternal short-axis of the right ventricular outflow tract, showing mild pulmonary stenosis.

Additionally, moderate pulmonic regurgitation with increased flow velocity (2.3 m/s) and an elevated gradient (21.2 mmHg) through the pulmonic annulus was observed (Video 3, Figure 1). The right atrium was markedly dilated, and a small anterior pericardial effusion was identified. An electrocardiogram revealed right axis deviation and an incomplete right bundle branch block (RBBB).

**Video 3:** Parasternal short-axis of the right ventricular outflow tract, exemplifying turbulent flow through the pulmonary valve with moderate regurgitation.

Given the patient’s asymptomatic status, treatment with torsemide was initiated, and a follow-up TTE was planned in three months. Further diagnostic testing, including a 5-hydroxyindoleacetic acid (5-HIAA) 24-hour urine collection and abdominal ultrasound, was also ordered.

**Figure 2:**
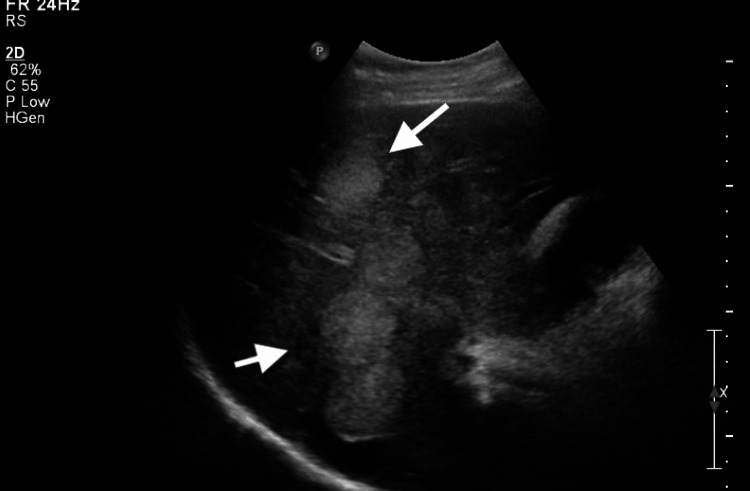
Ultrasound of the liver demonstrating multiple hypoechoic masses in the left hepatic lobe (white arrows).

**Figure 3:**
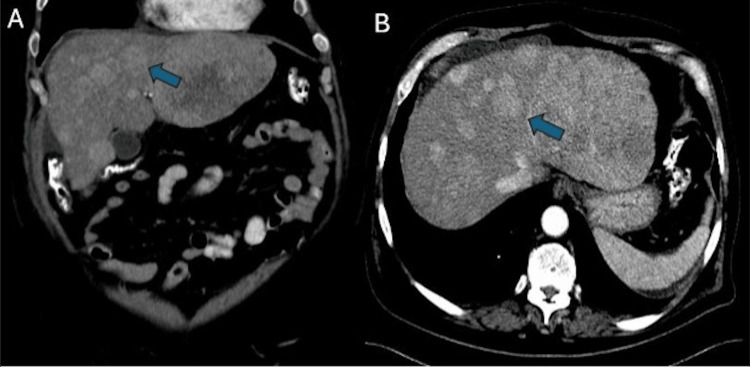
CT scan of the abdominal cavity showing widely spread heterogenous metastasis of the neoplasm to the liver (blue arrows). A) Coronal plane CT scan with contrast; B) transverse plane CT scan with contrast

At the three-month follow-up, the 5-HIAA urine collection revealed significantly elevated levels at 499.3 mg/24 hours (normal range: 2-9 mg/24 hours). Abdominal ultrasound demonstrated a heterogeneously enlarged liver with multiple hypoechoic masses, including a dominant mass in the left hepatic lobe (Figure 2). Based on these findings, a carcinoid tumor was suspected, prompting a CT scan of the abdomen and pelvis. The CT scan showed numerous metastatic lesions throughout the liver and a mesenteric mass adherent to the small bowel with calcifications (Figure 3).

A CT-guided biopsy of a hepatic lesion confirmed a well-differentiated grade-3 neuroendocrine carcinoma. The patient was referred to an oncologist and started on lanreotide. A combination regimen of capecitabine and temozolomide was subsequently added. Given the diagnosis of metastatic carcinoid tumor and associated CHD, the patient was referred to a tertiary care center for specialized management.

Two months later, the patient developed shortness of breath on exertion and 1-2+ lower extremity edema. During this period, he also developed new-onset ascites, requiring multiple paracenteses for symptom control. As the disease progressed, symptoms consistent with heart failure emerged, necessitating surgical intervention.

The patient underwent tricuspid and pulmonary valve replacements with 31 mm and 27 mm Magna Ease bioprosthetic valves (Edwards Lifesciences, Santa Ana, CA), respectively. A post-surgical echocardiogram revealed an LVEF of 65%, mild-to-moderate tricuspid regurgitation, resolved pulmonary regurgitation, right ventricular hypertrophy, and mildly decreased right ventricular systolic function (Video 4 and Video 5).

**Video 4:** Post-valvular replacement (pulmonic and tricuspid valve) apical four-chamber view of a well-functioning 31 mm Magna Ease bioprosthetic valve, showing normal opening and coaptation.

**Video 5:** Post-valvular replacement parasternal short-axis of the right ventricular outflow tract, showing a well-functioning 27 mm Magna Ease bioprosthetic valve, with Doppler scan exemplifying normal linear flow and absence of regurgitant flow.

Following surgery, the patient experienced complete resolution of heart failure symptoms. He required two additional paracenteses before achieving the resolution of ascites. At present, the patient remains in remission with ongoing stability in both oncologic and cardiac outcomes.

## Discussion

CHD is a rare but impactful cardiac complication arising from metastatic NETs. The interplay of bioactive substances, such as serotonin and other vasoactive agents, leads to characteristic fibrotic changes predominantly in the right-sided heart valves [9]. Several case studies have emphasized the varying manifestations of CHD, emphasizing the hallmark fibrotic thickening and retraction of tricuspid and pulmonary valves, which can culminate in severe right heart failure [9,10].

Most patients present with a complaint of dyspnea in the setting of hypovolemia and are found to have a murmur on examination [11-12]. A previous study of patients with verified CHD found that tricuspid regurgitation murmur was present in 77% of patients. The study found that 32% of patients had a pulmonary valve stenosis murmur, 31% had a pulmonary regurgitation murmur, 4% had incidentally found left-sided murmurs, and 8% presented without a murmur [11]. The hallmark of CHD is the endocardial fibrosis of the tricuspid heart valve, its associated structures, and, to a minor degree, fibrosis of the pulmonary valve [12]. The valvular fibrosis of leaflets, chordae tendineae, and the associated papillary muscles initially leads to fibrotic thickening and progresses to valvular leaflet fixation and retraction, resulting in tricuspid valve annular dilatation [13]. In our patient, tricuspid regurgitation and pulmonary stenosis murmurs were detected upon auscultation, aligning with physical examination findings common in CHD patients. Dyspnea was absent at presentation, suggesting early disease stage. This supports evidence that cardiac symptoms evolve with prolonged exposure to circulating vasoactive substances. Furthermore, our patient also demonstrates a right axis deviation and an incomplete RBBB, as seen in the electrocardiogram; this finding generally accounts for only 11% to 30% of cases [14,15]. 

Typically, only carcinoid tumors that metastasize to the liver result in pathological changes to the heart [14]. This phenomenon is attributed to the paraneoplastic effects of vasoactive substances, including 5-hydroxytryptamine (5-HT), commonly referred to as serotonin, and other vasoactive compounds [14]. These substances are generally inactivated by the liver and lungs; however, the incidence of hepatic metastasis permits significant quantities of these substances to bypass hepatic inactivation and reach the right side of the heart [16]. This process will cause endocardial plaques of fibrous tissue formation, not just in the valve leaflets but also in the subvalvular apparatus, including the tendinous chords and papillary muscle. Although the fibrous tissue may distort the valves, the morphology of the valve leaflets is usually not disrupted. However, endocardial thickening may lead to valve retraction and fixation. 

Multiple studies support that cardiac involvement is proportional to circulating serotonin levels, linking severe diarrhea, flushing, and dyspnea to advanced valvular dysfunction [17-18]. This same pathology was seen on our patient with severe tricuspid valve regurgitation and fixation during ventricular contraction, severe pulmonary stenosis, and a right ventricular enlargement with endocardial fibrosis, causing the patient to develop more severe symptoms, including shortness of breath during exertion and dyspnea. The pathophysiology of this process is unclear; studies have implied that serotonin, a vasoactive substance secreted by NET, has fibroblast proliferative properties such as tachykinins or transforming growth factor-B, which lead to the deposition of plaques on the endocardial surfaces [19]. This rationale is based on mRNA expression for the serotonin receptors, especially the 5-HT2B serotonin receptor seen in the cardiovascular system [20]. 

Management of CHD emphasizes reducing serotonin levels and addressing valvular dysfunction. Current therapeutic strategies include pharmacotherapy with somatostatin analogs, which reduce serotonin secretion and systemic symptoms of carcinoid syndrome but are ineffective in reversing fibrotic changes [20]. Emerging therapies targeting 5-HT2B receptors, such as serotonin receptor antagonists, show potential in preventing further fibrosis by inhibiting serotonin-mediated fibroblast activation [12,20]. Surgical valve replacement remains the gold standard for addressing severe valve damage, particularly in patients with stable NET control [20]. Experimental approaches, including antifibrotic therapies targeting TGF-β pathways and serotonin transporter inhibitors, offer promise in mitigating fibrotic progression [20]. This case underscores the irreversible nature of CHD fibrosis and highlights the critical need for early detection, with surgical intervention providing an opportunity to reduce mortality by addressing severe valve dysfunction in a setting of right-sided heart failure.

## Conclusions

This case demonstrates the complex interplay of bioactive substances in CHD, highlighting the critical role of serotonin and tachykinin in fibrotic progression. The correlation between carcinoid syndrome symptoms and cardiac involvement underscores the importance of timely diagnosis in patients with NETs. While pharmacological treatments aim to control serotonin levels, surgical intervention remains essential for advanced valve damage. Continued advancements in antifibrotic therapies and serotonin receptor antagonists show promise in managing CHD by targeting pathways that reduce fibrosis and improve heart function. However, challenges such as variable efficacy, safety concerns, and the complexity of long-term treatment need to be addressed. Surgical valve replacement remains the most effective treatment for severe cases, offering improved survival and quality of life despite risks like perioperative complications and potential recurrence. A multidisciplinary approach is essential to combine emerging therapies with surgical expertise, improving outcomes and long-term management for patients with CHD.
